# Is there a causal role for homocysteine concentration in blood pressure? A Mendelian randomization study^1^,^2^

**DOI:** 10.3945/ajcn.115.116038

**Published:** 2015-12-16

**Authors:** Maria C Borges, Fernando P Hartwig, Isabel O Oliveira, Bernardo L Horta

**Affiliations:** 3 Postgraduate Program in Epidemiology and; 4 Department of Physiology and Pharmacology, Institute of Biology, Universidade Federal de Pelotas, Pelotas, Brazil

**Keywords:** blood pressure, cohort studies, homocysteine, Mendelian randomization, molecular epidemiology

## Abstract

**Background:** An understanding of whether homocysteine is a cause or a marker of increased blood pressure is relevant because blood homocysteine can be effectively lowered by safe and inexpensive interventions (e.g., vitamin B-6, B-9, and B-12 supplementation).

**Objective:** The aim was to assess the causal influence of homocysteine on systolic and diastolic blood pressure (SBP and DBP, respectively) in adults with the use of Mendelian randomization (MR).

**Design:** Data from the 1982 Pelotas Birth Cohort (Brazil) were used. A total of 4297 subjects were evaluated in 2004–2005 (mean age: 22.8 y). The association of homocysteine concentration with SBP and DBP was assessed by conventional ordinary least-squares (OLS) linear regression and 2-stage least-squares (2SLS) regression (MR analysis). The single nucleotide polymorphism (SNP) methylenetetrahydrofolate reductase (*MTHFR*) C677T (rs1801133) was used as proxy for homocysteine concentration. We also applied MR to data from the International Consortium for Blood Pressure (ICBP) genomewide association studies (>69,000 participants) using rs1801133 and additional homocysteine-associated SNPs as instruments.

**Results:** In OLS regression, a 1-SD unit increase in log homocysteine concentration was associated with an increase of 0.9 (95% CI: 0.4, 1.4) mm Hg in SBP and of 1.0 (95% CI: 0.6, 1.4) mm Hg in DBP. In 2SLS regression, for the same increase in homocysteine, the coefficients were −1.8 mm Hg for SBP (95% CI: −3.9, 0.4 mm Hg; *P* = 0.01) and 0.1 mm Hg for DBP (95% CI: −1.5, 1.7 mm Hg; *P* = 0.24). In the MR analysis of ICBP data, homocysteine concentration was not associated with SBP (β = 0.6 mm Hg for each 1-SD unit increase in log homocysteine; 95% CI: −0.8, 1.9 mm Hg) but was positively associated with DBP (β = 1.1 mm Hg; 95% CI: 0.2, 1.9 mm Hg). The association of genetically increased homocysteine with DBP was not consistent across different SNPs.

**Conclusion:** Overall, the present findings do not corroborate the hypothesis that homocysteine has a causal role in blood pressure, especially in SBP.

## INTRODUCTION

A higher risk of coronary artery disease is observed among subjects with very high plasma homocysteine concentrations, as in some rare genetic defects, including mutations in the gene encoding cystathionine β-synthase (CBS).^5^ This finding raised the hypothesis that homocysteine might be involved in the etiology of cardiovascular diseases (CVDs) (1, 2).

The association between homocysteine and CVD was initially replicated in retrospective studies, but subsequent prospective studies yielded considerably weaker associations (3, 4), raising the concern that the association of hyperhomocysteinemia with CVD risk in the general population could be explained by reverse causality or residual confounding (5). On the other hand, this association has biological plausibility, because homocysteine has been associated with increased blood pressure (6, 7).

The use of genetic variants as instruments to improve causal inference in observational studies is known as Mendelian randomization (MR). The term comes from Mendel’s laws of inheritance, stating that allele pairs separate to form gametes (first law) and that alleles in different loci segregate independently from each other during gamete formation (second law) (8). In contrast to traditional observational studies, MR is not as susceptible to confounding, measurement error, and reverse causality, because genotype is defined at conception, genetic variants can be precisely measured, and their distribution is usually independent of “classical” confounders such as socioeconomic and lifestyle characteristics, provided that there is no population stratification (9, 10).

MR has been used previously to investigate the potential causal role of homocysteine in coronary artery disease (11) and stroke (12) with the use of a single nucleotide polymorphism (SNP) in the gene encoding the enzyme 5,10-methylenetetrahydrofolate reductase (*MTHFR*) as an instrumental variable (IV) for circulating homocysteine concentration. This SNP, known as *MTHFR* C677T (rs1801133), consists of a cytosine to thymidine substitution at nucleotide 677 in *MTHFR*. This results in an alaline to valine substitution at codon 222 of the enzyme, which is detrimental for its activity and leads to an important increase in blood homocysteine concentrations. In a previous meta-analysis, the association between this SNP and coronary disease was modest or negligibly different from zero (11). The association with stroke varied according to geographical location, with null findings in European, North American, and Australian studies and positive findings in Asian studies. This heterogeneity might be attributed to effect modification by folate intake or to small-study bias (12).

An understanding of the causal role of hyperhomocysteinemia in vascular disease and its risk factors, including blood pressure, is relevant because homocysteine concentration can be effectively lowered by simple, safe, and inexpensive interventions, such as supplementation with folic acid, vitamin B-6, and vitamin B-12 (13, 14). This study’s aim was to assess the causal influence of homocysteine on blood pressure in adults with the use of the MR approach.

## METHODS

### Data sources

This study included individual-level data from the 1982 Pelotas Birth Cohort and summary data from the following different consortia: the International Consortium for Blood Pressure (ICBP) (15), the largest meta-analysis of genomewide association studies (GWASs) for homocysteine concentration available (16), the Global Lipids Genetics Consortium (GLGC) (17), the Meta-Analyses of Glucose and Insulin-Related Traits Consortium (MAGIC) (18, 19), and the Genetic Investigation of Anthropometric Traits (GIANT) Consortium (20, 21). Details about each data source are provided below.

### 1982 Pelotas Birth Cohort

#### Participants

Pelotas is a medium-sized city, with nearly 330,000 inhabitants, located in the south of Brazil. In 1982, all maternity hospitals in the city were visited daily, and 99.2% of the births were identified. Those live-born infants whose families lived in the urban area of the city were evaluated and their mothers interviewed (*n* = 5914). These subjects have been followed up on several occasions. Further details of the study methodology have been described elsewhere (22, 23). In 2004–2005, 4297 members of the cohort (mean age: 22.8 y; range: 21.9–23.7 y) were evaluated, which, when added to the 282 known to have died, represented a follow-up rate of 77.4%. The subjects answered a questionnaire and had venous blood samples collected.

#### Variables

Systolic and diastolic blood pressure (SBP and DBP, respectively) were measured at the beginning and at the end of the interview by using a calibrated digital wrist blood pressure monitor (Omron HEM-629) on the left arm. Before each measurement, the individual was instructed to sit and rest for at least 5 min. The mean of the 2 measurements was used. The circulating homocysteine concentration was determined by chemiluminescence immunoassay (24) with the use of the Immulite System (Siemens Health Care Diagnostics).

DNA was extracted from peripheral blood leukocytes by sequential lysis with the use of the salting-out technique, adapted from the protocol by Miller et al. (25). After extraction, DNA was frozen at −70°C. Genotyping was performed by using the Illumina HumanOmni2.5-8v1 array (Illumina Inc.). Quality-control exclusion criteria for SNPs consisted of a Hardy-Weinberg equilibrium *P* value <1 × 10^−7^, being monomorphic, and a genotyping rate <95%. For individuals, exclusion criteria were as follows: missing information for >3% of genotyped SNPs and cryptic relatedness [kinship >0.1, as described elsewhere (26)]. For this study, we used the SNP *MTHFR* C677T as an instrument due to its well-characterized and strong association with circulating homocysteine concentrations.

Covariates were as follows: sex (male or female), skin color (white, black, brown, or other), years of education (0–4, 5–8, 9–11, or ≥12 y), family income (quintiles of minimum wage units), regular alcohol intake (nondrinkers, <2 drinks/d, and ≥2 drinks/d; 1 drink = 15 g alcohol), leisure-time physical activity (<150 or ≥150 min/wk), current smoking (0, 1–10, 11–20, or >20 cigarettes/d), and BMI (in kg/m^2^; underweight: <18.5; normal weight: 18.5–24.9; overweight: 25–29.9; or obese: ≥30). Weight and height, used in BMI calculation, were measured by using standard criteria (27).

### Summary data

#### ICBP

The ICBP initiative conducted a meta-analysis of 29 GWASs for blood pressure including >69,000 individuals of European ancestry and 2.5 million genotyped or imputed SNPs (15). The analyses were performed by using an additive genetic model and were adjusted for age, age squared, BMI, antihypertensive medication use, study-specific variables, and genomic control inflation factor. We directly requested summary data on the association of SNPs with SBP and DBP from ICBP investigators.

#### Meta-analysis of GWASs for homocysteine concentration

The meta-analysis included data from 10 GWASs on homocysteine concentration with a total of 44,147 individuals of European ancestry. Analyses were performed on sex-specific and age-adjusted SD units of natural log-transformed homocysteine concentration and by using an additive genetic model. Genomic control was used in each cohort before the meta-analysis. We extracted data on the association of SNPs with homocysteine concentration directly from van Meurs et al. (16).

#### GLGC

The GLGC included 60 cohort and case-control studies with GWAS or Metabochip data and HDL-cholesterol, LDL-cholesterol, and triacylglycerol data for 188,577 individuals of European ancestry (17). Analyses were performed by using an additive genetic model and were adjusted for age, sex, study-specific variables, and genomic control inflation factor. Individuals taking lipid-lowering medications were excluded. Data on the association of SNPs with HDL cholesterol, LDL cholesterol, and triacylglycerol were downloaded from http://csg.sph.umich.edu/abecasis/public/lipids2013/.

#### MAGIC

MAGIC included 23 cohort studies with GWAS and glycated hemoglobin (HbA1c) data on 38,238 individuals of European ancestry (18) and 20 cohort studies with GWAS and fasting insulin data on 35,920 individuals of European ancestry (19). Analyses were performed by using an additive genetic model and were adjusted for age, sex, cohort-specific variables, and genomic control inflation factor (λ). Data on the association of SNPs with HbA1c and fasting insulin were downloaded from http://www.magicinvestigators.org/downloads/.

#### GIANT

GIANT included 114 studies of multiple designs with GWAS or Metabochip and BMI data on 38,238 individuals of European ancestry (20) and 101 studies of multiple designs with GWAS or Metabochip and waist circumference (WC) data on 210,088 individuals of European ancestry (21). BMI analyses were performed by using an additive genetic model and were adjusted for age, age squared, study-specific variables, and genomic control inflation factor (λ). WC analyses were adjusted for sex, BMI, study-specific variables, and genomic control inflation factor (λ). Data on the association of SNPs with BMI and WC were downloaded from http://www.broadinstitute.org/collaboration/giant/index.php/GIANT_consortium_data_files.

### Data analysis

#### 1982 Pelotas Birth Cohort

**FIGURE 1:**
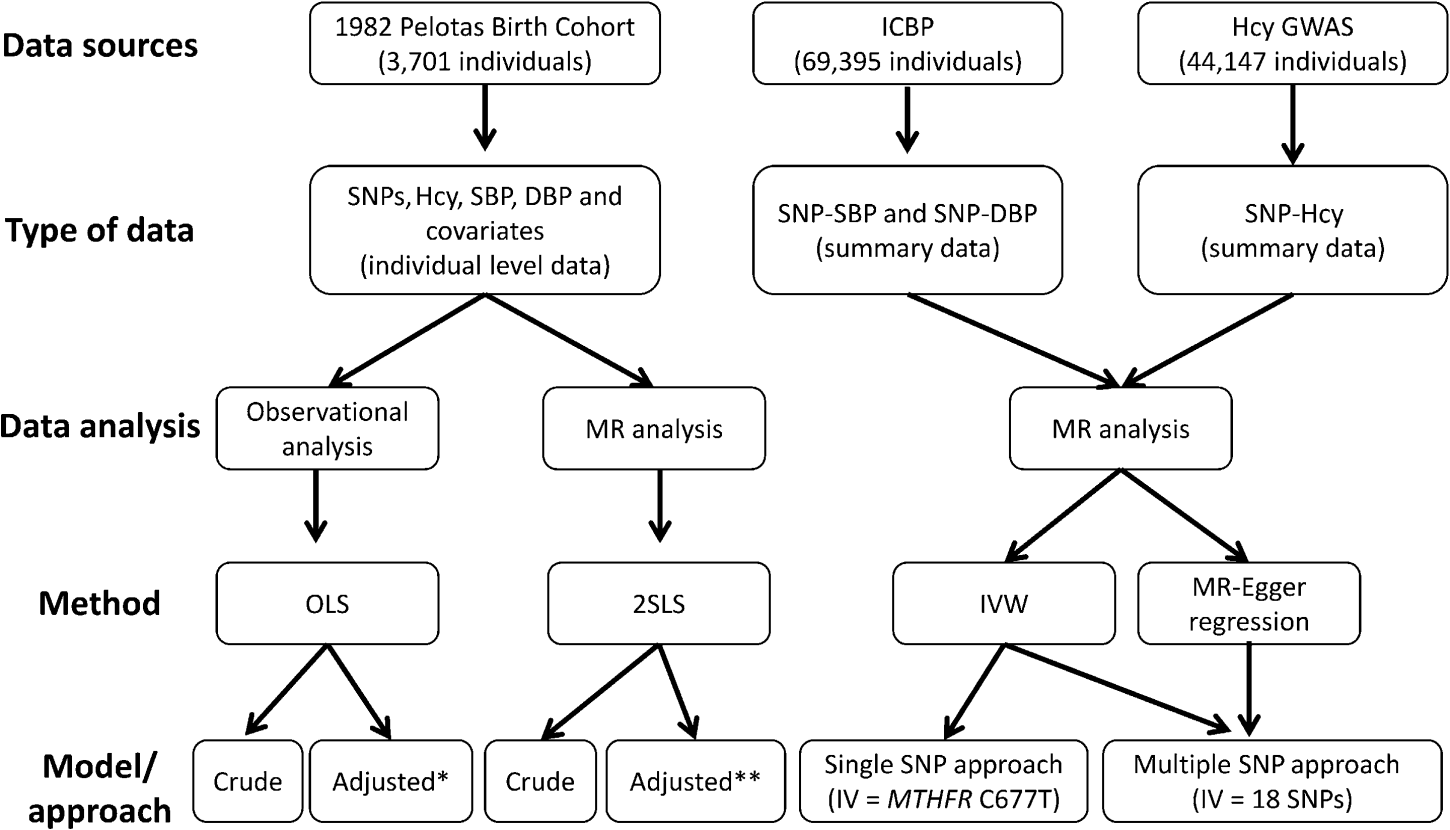
Analysis plan. Individual data from the 1982 Pelotas Birth Cohort were used to estimate the association of homocysteine concentration with SBP and DBP [unadjusted “crude” model and adjusted for potential confounders (“adjusted*” model)] and to investigate the association of genetically increased homocysteine concentration with SBP and DBP [MR analysis; unadjusted “crude” model and adjusted for genomic ancestry (“adjusted**” model)]. Summary data from the ICBP and homocysteine GWASs were combined to further investigate the association of genetically increased homocysteine concentration with SBP and DBP (MR analysis) by using as an IV the SNP *MTHFR* C677T (single SNP approach) or 18 SNPs associated with homocysteine concentration (multiple SNP approach). DBP, diastolic blood pressure; GWAS, genomewide association study; Hcy, homocysteine; ICBP, International Consortium for Blood Pressure; IV, instrumental variable; IVW, inverse variance weighted; MR, Mendelian randomization; *MTHFR*, methylenetetrahydrofolate reductase; OLS, ordinary least-squares linear regression; SBP, systolic blood pressure; SNP, single nucleotide polymorphism; 2SLS, 2-stage least-squares regression.

Multinomial regression models were used to verify if *MTHFR* C677T genotype distribution was associated with the covariates (sex, skin color, years of education, family income, regular alcohol consumption, leisure-time physical activity, smoking, and BMI). Homocysteine was log transformed, due to its positively skewed distribution, and standardized (SD units). Crude and adjusted (for sociodemographic and lifestyle variables) associations of standardized log homocysteine concentration with SBP and DBP were evaluated by using conventional ordinary least-squares (OLS) linear regression (**Figure 1**).

MR analysis of the association of standardized log homocysteine concentration with SBP and DBP was performed by using 2-stage least-squares (2SLS) regression, which is an IV estimation technique (28). In the first stage, homocysteine concentration is regressed against the IV. The second stage consists of regressing the values of SBP and DBP against the predicted values of the first model. The *MTHFR* C677T variant was coded in the additive genetic model according to the number of copies of the T allele. Results from OLS and 2SLS regression were compared by using the Durbin-Wu-Hausman (DWH) test (28–31) (Figure 1).

To control for population stratification, analyses were adjusted for the top 10 ancestry-informative principal components (calculated by using a linkage disequilibrium–pruned subset of 655,046 autosomal SNPs) (32).

#### Summary data

To further explore the potential causal effect of homocysteine concentration on SBP and DBP, we also analyzed summary data from the ICBP. All SNP effect alleles were harmonized to reflect homocysteine-increasing alleles.

Two approaches were applied to the ICBP data, here referred to as “single SNP” and “multiple SNP” approaches (Figure 1). In the single SNP approach, only the SNP *MTHFR* C677T (rs1801133) was used as an IV, which has a well-established functional role in homocysteine concentration. The IV β-coefficients and SEs were calculated by using the inverse variance weighted (IVW) method [described by Burgess et al. (33)], as follows:


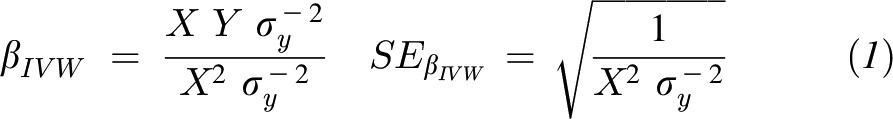


where *X* is the mean change in standardized log homocysteine concentration per additional effect allele of *MTHFR* C677T and *Y* is the mean change in SBP or DBP (mm Hg) per additional effect allele of *MTHFR* C677T with SE σ*_y_*.

In the multiple SNP approach, all SNPs associated with homocysteine concentration in the largest GWAS available (16) were used, regardless of evidence of a functional impact of the SNP on homocysteine concentration (*n* = 18 SNPs). Characteristics of these SNPs and their association with the studied phenotypes are summarized in **Supplemental Table 1**. Data from the 18 SNPs were used *1*) to estimate the average effect of the SNPs on homocysteine concentration, SBP, and DBP by using a fixed-effects meta-analysis model with inverse variance weights and the heterogeneity of the association across SNPs, measured by *I*^2^, and *2*) to investigate the effect of genetically increased homocysteine concentration on SBP and DBP (IV estimation).

The IV estimation for the multiple SNP approach was based on 2 methods. The first was the IVW method, in which IV estimates for each SNP (indexed by *k*) were combined in a fixed-effects meta-analysis model (33), as follows:


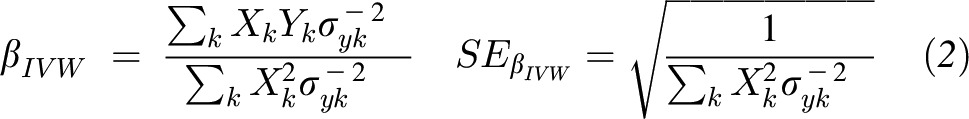


In case ≥1 SNPs influence SBP or DBP independently of homocysteine concentration (horizontal pleiotropy) and such direct effects do not cancel out, MR assumptions are violated and IV estimates from the IVW method will be biased. To account for horizontal pleiotropy, a second IV estimation method was used in the multiple SNP approach, the MR-Egger regression method, recently proposed by Bowden et al. (34).

The MR-Egger regression is an adaptation of the Egger regression, in which a regression model is fitted by using regression coefficients for SNP-outcome (SBP or DBP) associations as the dependent variable and regression coefficients for SNP-exposure (homocysteine) as the independent variable, weighting by the inverse variance of SNP-outcome associations. In this method, the intercept will reflect the average horizontal pleiotropic effect across genetic variants and the slope will be a valid causal effect estimate provided that the InSIDE (Instrument Strength Independent of Direct Effect) assumption holds, which requires that there is no correlation between SNP-exposure associations and direct effects of SNPs on the outcome. Bootstrapping (10,000 iterations) was used to derive corrected 95% CIs for MR-Egger intercept and slope by using the percentile method (34).

As a sensitivity analysis, to further explore the issue of horizontal pleiotropy, the same IVs (both single SNP and the multiple SNP approaches with the IVW method) were used to investigate the association of genetically increased homocysteine concentration with the following phenotypes: HDL cholesterol, LDL cholesterol, triacylglycerol, fasting insulin, HbA1c, BMI, and WC.

All analyses were performed with Stata 12.1 software (StataCorp). Stata ivregress command was used for 2SLS regression models (35).

### Ethical issues

All phases of the 1982 Pelotas Birth Cohort Study were approved by the Research Ethics Committee of the Federal University of Pelotas, which is affiliated with the Brazilian Federal Medical Council. Written informed consent was obtained from participating subjects in the 2004–2005 visit.

## RESULTS

### 1982 Pelotas Birth Cohort

A total of 3701 individuals had complete genotyping, blood pressure, and biochemical data. Most individuals self-reported as white, had completed 9–11 y of formal education, drank up to 2 drinks of alcohol/d, were inactive during leisure time, were nonsmokers, or had a normal BMI (**Table 1**).

**TABLE 1:** Sociodemographic and lifestyle characteristics of participants from the 1982 Pelotas Birth Cohort, 2004–2005

	*n* (%)
Sex	
Male	1860 (50.3)
Female	1841 (49.7)
Skin color	
White	2768 (74.8)
Black	597 (16.1)
Brown	196 (5.3)
Other	140 (3.8)
Years of education	
0–4	302 (8.2)
5–8	1042 (28.2)
9–11	1817 (49.1)
≥12	540 (14.6)
Family income, quintile	
1 (poorest)	774 (20.9)
2	754 (20.4)
3	723 (19.5)
4	712 (19.2)
5 (wealthiest)	738 (19.9)
Alcohol consumption	
Nondrinkers	1203 (32.5)
<2 drinks/d	1864 (50.4)
≥2 drinks/d	634 (17.1)
Leisure-time physical activity	
Inactive (<150 min/wk)	2595 (70.1)
Active (≥150 min/wk)	1106 (29.9)
Smoking	
Nonsmokers	2745 (74.2)
1–10 cigarettes/d	513 (13.9)
11–20 cigarettes/d	375 (10.1)
>20 cigarettes/d	68 (1.8)
BMI	
Underweight	216 (5.8)
Normal weight	2407 (65.1)
Overweight	770 (20.8)
Obese	305 (8.3)
Total	3701 (100.0)

The *MTHFR* C677T SNP was in Hardy-Weinberg equilibrium (*P* = 0.49) and was associated with skin color (*P* < 0.001), but this association disappeared after adjustment for the top 10 ancestry-informative principal components (*P* = 0.91). None of the covariates tested were associated with the *MTHFR* C677T variant after this adjustment (**Table 2**).

**TABLE 2:** Genotype distribution according to covariates in the 1982 Pelotas Birth Cohort, 2004–2005

		rs1801133, %				
	*n*	CC	CT	TT	*P*^1^	*P*^2^
Sex					0.13	0.09
Male	1860	49.6	41.6	8.8		
Female	1841	46.3	44.3	9.4		
Skin color					<0.001	0.91
White	2768	44.8	45.3	9.8		
Black	597	60.8	33.2	6.0		
Brown	196	54.1	38.8	7.1		
Other	140	45.7	43.6	10.7		
Years of education					0.24	0.85
0–4	302	48.3	41.7	9.9		
5–8	1042	49.5	41.6	8.9		
9–11	1817	48.5	42.5	8.9		
≥12	540	42.6	47.8	9.6		
Family income, quintile					0.23	0.57
1 (poorest)	774	47.7	42.6	9.7		
2	754	52.5	39.9	7.6		
3	723	47.4	43.7	8.9		
4	712	46.8	43.4	9.8		
5 (wealthiest)	738	45.1	45.3	9.6		
Alcohol consumption					0.70	0.81
Nondrinkers	1203	49.3	41.3	9.4		
<2 drinks/d	1864	47.1	43.9	9.1		
≥2 drinks/d	634	48.0	43.4	8.7		
Leisure-time physical activity					0.20	0.45
Inactive (<150 min/wk)	2595	44.0	44.8	11.2		
Active (≥150 min/wk)	1106	48.3	42.8	8.9		
Smoking					0.27	0.59
Nonsmokers	2745	47.3	43.7	9.0		
1–10 cigarettes/d	513	52.5	37.6	9.9		
11–20 cigarettes/d	375	45.9	44.8	9.3		
>20 cigarettes/d	68	47.0	45.6	7.4		
BMI					0.73	0.76
Underweight	216	48.6	42.6	8.8		
Normal weight	2407	47.5	43.6	8.9		
Overweight	770	49.2	40.5	10.3		
Obese	305	47.9	44.3	7.9		
Total	3701	47.9	43.0	9.1		

1 *P* values for heterogeneity (multinomial logistic regression).

2 *P* values adjusted for the top 10 ancestry-informative principal components (multinomial logistic regression).

Men had higher concentrations of homocysteine (9.0 μmol/L; 95% CI: 8.9, 9.2 μmol/L) and higher SBP (123.4 mm Hg; 95% CI: 122.8, 124.1 mm Hg) and DBP (75.6 mm Hg; 95% CI: 75.0, 76.1 mm Hg) than did women (homocysteine: 7.1 μmol/L; 95% CI: 7.0, 7.2 μmol/L; SBP: 111.2 mm Hg; 95% CI: 110.6, 111.8 mm Hg; and DBP: 71.2 mm Hg; 95% CI: 70.7, 71.7 mm Hg). The following characteristics were also positively associated with homocysteine concentration and SBP and/or DBP: low educational level, high alcohol intake, and being physically active. Smoking was positively associated with homocysteine concentration and negatively with DBP. Family income, skin color, and BMI were associated with SBP and DBP but not with homocysteine concentration (**Table 3**).

**TABLE 3:** Homocysteine, SBP, and DBP according to covariables in the 1982 Pelotas Birth Cohort, 2004–2005^1^

	*n*	Homocysteine,^2^ μmol/L	SBP, mm Hg	DBP, mm Hg
Sex				
Male	1860	9.0 (8.9, 9.2)	123.4 (122.8, 124.1)	75.6 (75.0, 76.1)
Female	1841	7.1 (7.0, 7.2)	111.2 (110.6, 111.8)	71.2 (70.7, 71.7)
Skin color				
White	2768	8.0 (7.9, 8.1)	116.9 (116.4, 117.5)	73.3 (72.9, 73.7)
Black	597	8.0 (7.8, 8.2)	119.0 (117.7, 120.2)	74.1 (73.1, 75.1)
Brown	196	8.0 (7.6, 8.4)	118.5 (116.4, 120.6)	73.4 (71.6, 75.2)
Other	140	8.1 (7.7, 8.6)	117.9 (115.3, 120.4)	72.7 (70.8, 74.6)
Years of education				
0–4	302	8.6 (8.3, 9.0)	117.5 (115.8, 119.2)	72.9 (71.6, 74.3)
5–8	1042	8.2 (8.0, 8.4)	118.0 (117.0, 118.9)	72.9 (72.2, 73.7)
9–11	1817	7.8 (7.7, 7.9)	117.6 (116.9, 118.3)	73.9 (73.3, 74.4)
≥12	540	8.0 (7.8, 8.2)	115.4 (114.2, 116.5)	73.0 (72.2, 73.8)
Family income, quintile				
1 (poorest)	774	8.1 (7.9, 8.3)	116.2 (115.1, 117.2)	72.7 (71.9, 73.6)
2	754	8.0 (7.8, 8.2)	117.6 (116.5, 118.7)	73.5 (72.6, 74.3)
3	723	7.9 (7.7, 8.1)	117.4 (116.3, 118.6)	73.3 (72.5, 74.1)
4	712	8.0 (7.8, 8.1)	118.1 (117.0, 119.1)	73.7 (72.9, 74.5)
5 (wealthiest)	738	8.1 (7.9, 8.3)	117.7 (116.6, 118.7)	73.8 (73.0, 74.6)
Alcohol consumption				
Nondrinkers	1203	7.7 (7.6, 7.9)	115.7 (114.9, 116.5)	73.2 (72.6, 73.9)
<2 drinks/d	1864	8.0 (7.9, 8.1)	117.1 (116.5, 117.8)	73.3 (72.9, 73.8)
≥2 drinks/d	634	8.7 (8.4, 8.9)	121.9 (120.8, 123.0)	74.8 (73.9, 75.6)
Leisure-time physical activity				
Inactive (<150 min/wk)	2595	7.8 (7.7, 7.9)	116.1 (115.6, 116.6)	73.8 (72.8, 74.9)
Active (≥150 min/wk)	1106	8.5 (8.3, 8.6)	120.7 (119.9, 121.6)	73.5 (73.2, 73.9)
Smoking				
Nonsmokers	2745	7.9 (7.8, 8.0)	117.5 (116.9, 118.0)	73.9 (73.4, 74.3)
1–10 cigarettes/d	513	8.2 (7.9, 8.4)	116.9 (115.7, 118.2)	72.5 (71.5, 73.5)
11–20 cigarettes/d	375	8.8 (8.5, 9.1)	116.9 (115.4, 118.5)	71.3 (70.1, 72.4)
>20 cigarettes/d	68	8.5 (7.7, 9.3)	119.8 (115.7, 123.8)	73.4 (70.4, 76.3)
BMI				
Underweight	216	8.2 (7.9, 8.6)	110.1 (108.2, 111.8)	69.5 (68.1, 70.9)
Normal weight	2407	8.0 (7.9, 8.1)	115.6 (115.1, 116.2)	72.1 (71.7, 72.5)
Overweight	770	8.1 (7.9, 8.3)	121.4 (120.3, 122.5)	75.8 (75.0, 76.7)
Obese	305	8.0 (7.7, 8.3)	126.1 (124.2, 128.0)	80.1 (78.6, 81.5)
Total	3701	8.0 (7.9, 8.1)	117.4 (116.9, 117.8)	73.4 (73.0, 73.8)

1 Values are means with 95% CIs in parentheses unless otherwise indicated. DBP, diastolic blood pressure; SBP, systolic blood pressure.

2 Values are geometric means of log homocysteine concentration.

**FIGURE 2:**
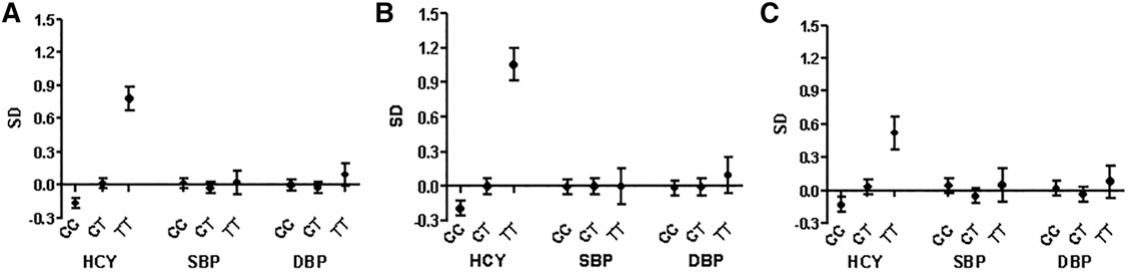
Standardized mean (95% CI) values for log homocysteine, SBP, and DBP according to genotypes of *MTHFR* C677T (rs1801133) in men and women combined (A), men (B), and women (C): 1982 Pelotas Birth Cohort, 2004–2005. Values were adjusted for the top 10 ancestry-informative principal components. A total of 1774 individuals had the CC genotype (922 men and 852 women), 1590 had the CT genotype (774 men and 816 women), and 337 had the TT genotype (164 men and 173 women). DBP, diastolic blood pressure; HCY, homocysteine; SBP, systolic blood pressure.

The *MTHFR* C677T SNP explained 5.3% of the variance in homocysteine concentration (*F* statistic = 208, *P* = 6 × 10^−46^). The T allele was associated with higher homocysteine concentrations in both sexes. Men with TT and CT genotypes had, on average, a 1.3- and a 0.2-SD higher log homocysteine concentration, respectively, compared with men with the CC genotype. In women, this difference was ∼ 0.7 and 0.2 SDs. There was no clear evidence of an association of *MTHFR* C677T with SBP or DBP (**Figure 2**).

**FIGURE 3:**
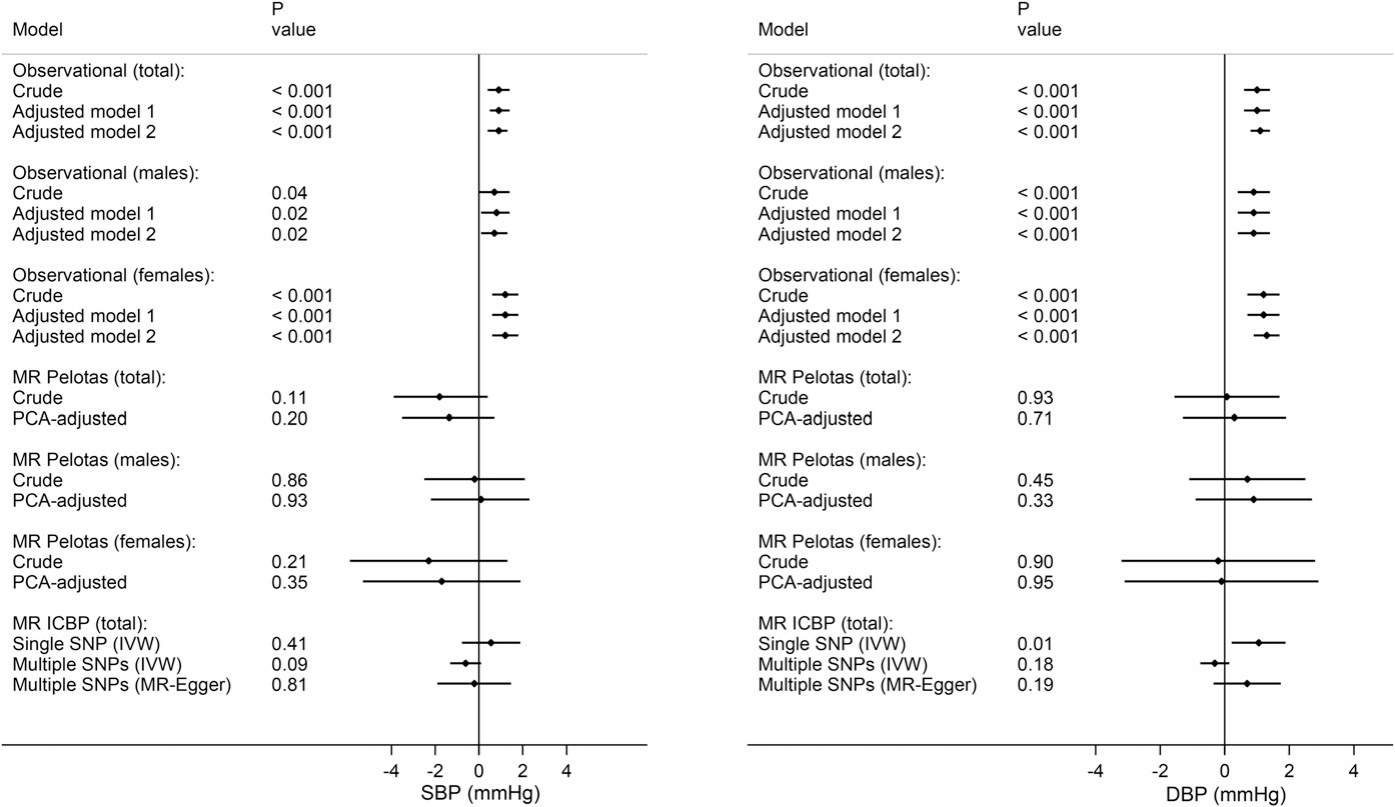
Mean difference in SBP and DBP per standardized unit of log homocysteine. Observational results were estimated by using (ordinary least-squares) linear regression (*n* = 3701 individuals from the Pelotas Birth Cohort) [crude model, adjusted for sociodemographic variables (adjusted model 1), and adjusted for sociodemographic and lifestyle variables (adjusted model 2)]. MR results were estimated for individual data from the 1982 Pelotas Birth Cohort (*n* = 3701 young adults) by using 2-stage least-squares regression [crude and adjusted for principal components of genomic ancestry (PCA-adjusted)] and for summary data from the ICBP (*n* >69,000 adults) by using the IVW method for both the single SNP and the multiple SNP approaches and the MR-Egger regression method for the multiple SNP approach only. DBP, diastolic blood pressure; ICBP, International Consortium for Blood Pressure; IVW, inverse variance weighted; MR, Mendelian randomization; PCA, principal components analysis; SBP, systolic blood pressure; SNP, single nucleotide polymorphism.

In unadjusted OLS regression, a 1-SD unit increase in log homocysteine concentration was associated with mean increases of 0.9 mm Hg (95% CI: 0.4, 1.4 mm Hg) in SBP and of 1.0 mm Hg (95% CI: 0.6, 1.4 mm Hg) in DBP. In the sex-specific analysis, the coefficients were 0.7 mm Hg (95% CI: 0.0, 1.4 mm Hg) for SBP and 0.9 mm Hg (95% CI: 0.3, 1.4 mm Hg) for DBP among men and 1.2 mm Hg (95% CI: 0.6, 1.8 mm Hg) for SBP and 1.2 mm Hg (95% CI: 0.7, 1.7 mm Hg) for DBP among women. Adjustment for socioeconomic and lifestyle variables did not substantially change these estimates (**Figure 3**).

According to the IV analysis of individual-level data (2SLS regression), the coefficients for a 1-SD increase in log homocysteine concentration were −1.8 mm Hg (95% CI: −3.9, 0.4 mm Hg) for SBP and 0.1 mm Hg (95% CI: −1.5, 1.7 mm Hg) for DBP. In the sex-specific analysis, the coefficients were −0.2 mm Hg (95% CI: −2.5, 2.1 mm Hg) in men and −2.3 mm Hg (95% CI: −5.9, 1.3 mm Hg) in women for SBP and 0.7 mm Hg (95% CI: −1.1, 2.5 mm Hg) in men and −0.2 mm Hg (95% CI: −3.1, 2.8 mm Hg) in women for DBP. Adjustment for ancestry-informative principal components slightly changed these estimates (Figure 3). None of the results from MR analysis of the Pelotas data were significantly different from zero. With regard to the comparison between regression coefficients from OLS and 2SLS, there was some evidence that the coefficients differed for SBP when considering the whole sample (*P* = 0.01, DWH test) but not for DBP (*P* = 0.24, DWH test).

### Summary data

**FIGURE 4:**
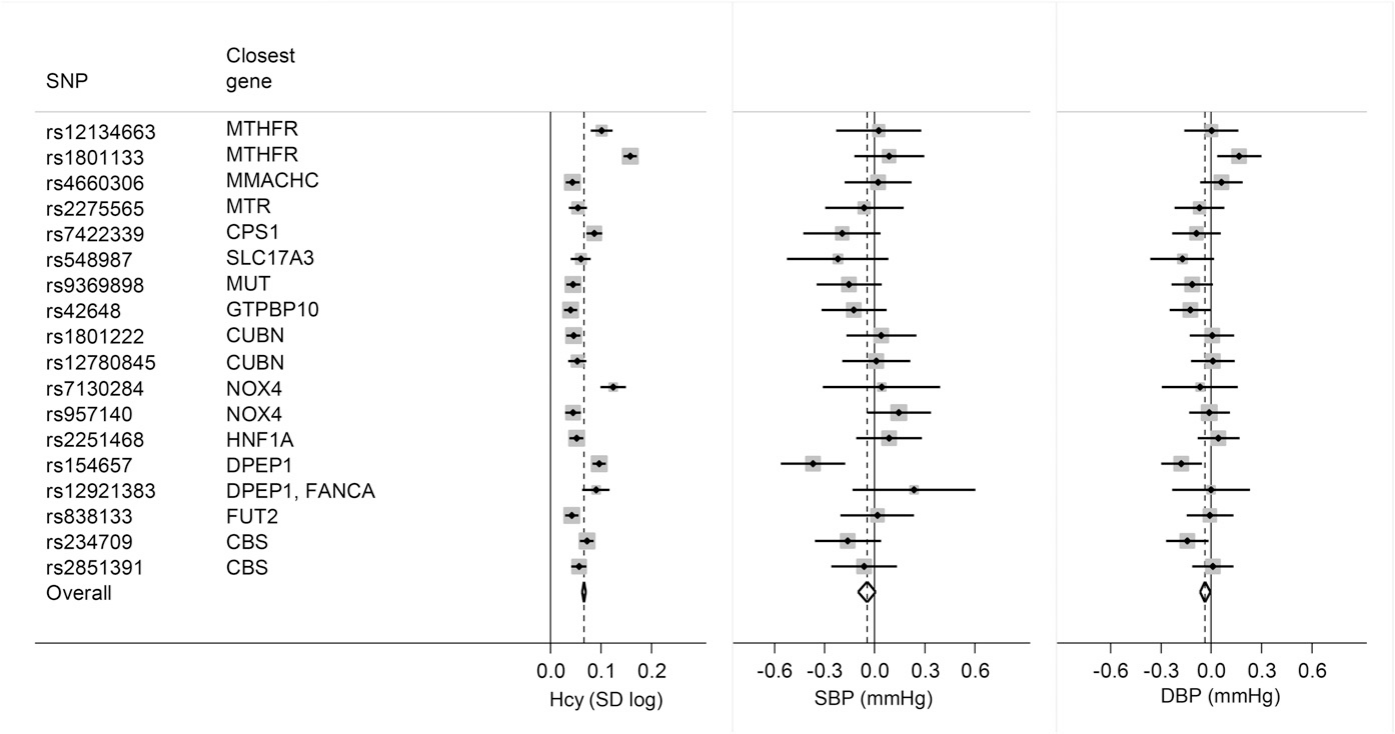
Forest plot of the mean difference in homocysteine concentration, SBP, and DBP per effect allele of 18 SNPs. The effect from all SNPs was combined into an overall effect by using fixed-effects meta-analysis (with inverse variance weights). Summary data were extracted from the largest homocysteine genomewide association study available (16) and from the International Consortium for Blood Pressure (15). CBS, cystathionine beta-synthase; CPS1, carbamoyl-phosphate synthase 1; CUBN, cubilin; DBP, diastolic blood pressure; DPEP1, dipeptidase 1; FANCA, Fanconi anemia complementation group A; FUT2, fucosyltransferase 2; GTPBP10, GTP-binding protein 10; Hcy, homocysteine; HNF1A, HNF1 homeobox A; MMACHC, methylmalonic aciduria cblC type; MTHFR, methylenetetrahydrofolate reductase; MTR, 5-methyltetrahydrofolate-homocysteine methyltransferase; MUT, methylmalonyl-CoA mutase; NOX4, NADPH oxidase 4; SBP, systolic blood pressure; SLC17A3, solute carrier family 17 member 3; SNP, single nucleotide polymorphism.

The analysis of ICBP data indicated that the SNPs rs154657 and rs234709 were negatively associated with SBP and/or DBP. The SNP rs1801133 (*MTHFR* C677T) was the only SNP that was positively associated with DBP. Heterogeneity across SNPs was high for the association with homocysteine concentration (*I*^2^ = 94%, *P* < 0.001) and moderate for the association with SBP (*I*^2^ = 40%, *P*-heterogeneity = 0.04) and DBP (*I*^2^ = 41%, *P* = 0.04) (**Figure 4**).

In the MR analysis using the *MTHFR* C677T variant as the IV (single SNP approach), homocysteine was not associated with SBP (β = 0.6 mm Hg/SD log homocysteine; 95% CI: −0.8, 1.9 mm Hg/SD log homocysteine) but was positively associated with DBP (β = 1.1 mm Hg/SD log homocysteine; 95% CI: 0.2, 1.9 mm Hg/SD log homocysteine). When the 18 SNPs were combined in the multiple SNP approach, genetically increased homocysteine concentration was not associated with SBP or DBP in either the IVW (SBP: β = −0.6 mm Hg; 95% CI: −1.3, 0.1 mm Hg; DBP: β = −0.3 mm Hg; 95% CI: −0.8, 0.1 mm Hg) or in the MR-Egger regression method (SBP: β = −0.2 mm Hg; 95% CI: −1.9, 1.5 mm Hg; DBP: β = 0.7 mm Hg; 95% CI: −0.4, 1.7 mm Hg) (Figure 3).

**FIGURE 5:**
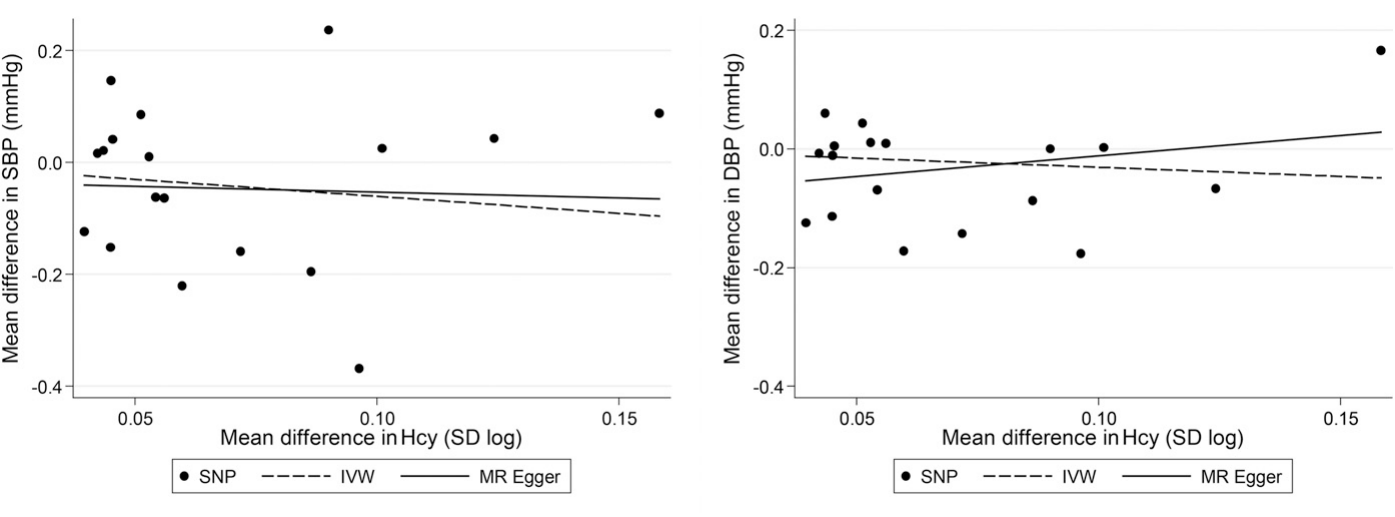
Scatter plot of the difference in SBP and DBP according to homocysteine concentration (*n* = 18 SNPs) estimated by using data from the International Consortium for Blood Pressure. Each data point represents βs for SNP–blood pressure (*y* axis) and SNP-homocysteine (*x* axis) associations. The fitted lines were derived from the IVW method (dashed line) and from the MR-Egger regression method (solid line). DBP, diastolic blood pressure; Hcy, homocysteine; IVW, inverse variance weighted; MR, Mendelian randomization; SBP, systolic blood pressure; SNP, single nucleotide polymorphism.

**Figure 5** shows that there was no dose-response relation between the effect of the SNPs on the outcomes (SBP or DBP) or on homocysteine concentration. In addition, the intercepts from the MR-Egger regression method provided no clear evidence of an effect of the SNPs on SBP or DBP independently of homocysteine concentration [intercept: −0.03 (95% CI: −0.15, 0.09) mm Hg for SBP and −0.08 (95% CI: −0.16, 0.00) mm Hg for DBP], indicating that it is unlikely that findings from the multiple SNP approach (especially for SBP) could be explained by horizontal pleiotropy (Figures 3 and 5).

In the MR analysis of other traits (blood lipids and glycemic and anthropometric traits), no association was observed when the SNP *MTHFR* C677T was used as the IV; however, when the multiple SNP approach was considered, there was a positive association with LDL cholesterol (**Supplemental Figure 1**).

## DISCUSSION

Overall, our findings supported a positive association between homocysteine concentration and blood pressure in young adults in the conventional observational analysis. However, in the MR analysis, there was no compelling evidence that genetically increased homocysteine concentration was associated with blood pressure, especially for SBP.

Similar to previous studies (7, 36), our findings from conventional regression analysis support that homocysteine concentration is positively associated with SBP and DBP among young adults. This association is biologically plausible. Some of the mechanisms by which homocysteine could influence blood pressure include oxidative stress, inflammation, and inhibition of nitric oxide synthesis (37–39), which might result in arterial stiffening and impaired endothelium-dependent vasodilation (40–42). However, because homocysteine concentration is associated with sociodemographic, lifestyle, and metabolic characteristics, it is difficult to conclude from conventional observational analyses whether homocysteine is a cause or just a marker of risk of CVDs.

Early randomized controlled trials showed that homocysteine-lowering interventions could improve blood pressure (40, 43, 44). However, findings from larger trials did not provide evidence of any improvement in SBP and DBP after folic acid supplementation, even though homocysteine concentration was substantially decreased (45–47).

To improve causal inference in the homocysteine–blood pressure association, we used MR to evaluate whether a genetic variant that is functionally associated with higher concentrations of homocysteine is also associated with blood pressure. MR studies explore the fact that the segregation of alleles during meiosis is analogous to the randomization process in randomized controlled trials, with the advantage that the “randomization of alleles” occurs at conception and thus reflects life-long exposure to a risk factor (e.g., increased homocysteine concentrations).

The MR analysis of individual-level data from 3701 Brazilian young adults did not provide clear evidence of a causal role of homocysteine concentration in SBP and DBP. Moreover, the DWH test provided evidence for a difference between the OLS and 2SLS estimates for SBP, suggesting that the OLS estimate was an overestimate of the causal effect (possibly due to residual confounding). However, because of large CIs resulting from the uncertainty inherent to IV analysis and our sample size, these results were not conclusive.

MR analysis of ICBP data, including >69,000 older adults, indicated no clear evidence of an association of homocysteine with SBP but a possible positive effect of homocysteine concentration on DBP. However, it is important to emphasize that this finding was largely influenced by the *MTHFR* C677T SNP and was not consistent across the other homocysteine-associated SNPs.

MR is a powerful tool for causal inference provided that the following assumptions are met: *1*) the genetic variant should be associated with the exposure of interest, *2*) it should be independent of exposure-outcome confounders, and *3*) it should affect the outcome only through the exposure (48). With regard to the first assumption, a strong IV is essential to reduce imprecision and, especially, to avoid weak instrument bias in MR analysis (49–51). In a one-sample MR setting, in which all information about the exposure and the outcome comes from the same sample (as in the analysis with data from the Pelotas cohort), weak instruments tend to bias the estimates toward the observational estimate (OLS). In our 2-sample MR setting (first sample: homocysteine GWASs; second sample: ICBP), in the presence of weak instrument bias the MR estimates would likely to be biased toward the null, because the 2 samples only partially overlapped (<22% of the ICBP participants were part of homocysteine GWASs) (52). In the one-sample MR, our IV (*MTHFR* C677T) was associated with homocysteine concentration, with *R*^2^ = 5.3% and an *F* statistic = 208 (*P* = 6 × 10^−46^; crude model), indicating that weak instrument bias is unlikely to be substantially influencing our analyses by using data from Pelotas cohort. In the 2-sample MR, only SNPs associated with homocysteine concentration at genomewide significance levels (*P* < 5 × 10^−8^) were included in the analyses (16).

Because the Pelotas sample is multiethnic and highly admixed, there could be confounding due to population stratification. From all covariates considered, only skin color was associated with the SNP, and this association was completely attenuated after adjustment for ancestry-informative principal components. This adjustment is known to be an efficient strategy to control for population stratification bias (32). Notably, this adjustment did not substantially change the strength of the associations. Both ICBP and homocysteine GWASs were restricted to individuals of European ancestry and used procedures to control for population structure (15, 16).

MR assumptions could also be violated in the case of horizontal pleiotropy (i.e., the genetic variant affects the outcome through pathways not mediated by the exposure or the genetic variant is in linkage disequilibrium with another variant that itself has pleotropic effects on the outcome). Although the presence of pleiotropy cannot be entirely ruled out, we addressed that using 2 main approaches: *1*) we restricted our analyses to one SNP (rs1801133) with well-described functional roles in homocysteine metabolism but known to influence other phenotypes, such as folate, that might influence the outcomes independently (53) and *2*) we broadened our analysis to all SNPs associated with homocysteine concentration in the largest GWASs available, regardless of knowledge about their functional impact, which allowed us to investigate if the potential influence of homocysteine on the outcomes was consistent across different SNPs. For the second approach, we used a recently developed method, the MR-Egger regression (34), which can assess and account for (at the cost of reducing statistical power) horizontal pleiotropic effects of genetic variants under the InSIDE assumption.

One further issue that should be considered is that, for summary data analysis only, information for SNP-homocysteine and SNP–blood pressure came from different sources. This approach, known as 2-sample MR, assumes that the 2 samples came from comparable populations (54). The samples from both the homocysteine GWAS consortium (16) and the ICBP (15) partially overlapped and were comparable in terms of genomic ancestry (all European-ancestry individuals from the United States and Europe). The proportion of women was higher in the homocysteine GWASs (82%) compared with ICBP data (most studies had 50–60% women). In both consortia, the majority of countries had not implemented folic acid fortification policies by the time of data collection (55).

As mentioned before, it is impossible to empirically completely rule out that MR results are not being driven by violations of IV assumptions. In this regard, it has been proposed that null findings from MR may be more reliable than non-null findings based on the logic that it is less likely that IV violations would perfectly balance each other out, because there is only one null value whereas there are infinite non-null possibilities (56). Although DBP results were inconclusive, findings for SBP consistently suggested the absence of a (strong) causal effect across the different approaches, including evidence for a difference between OLS and 2SLS in a 1-sample setting (in which weak instrument bias tends to approximate these estimates) (52).

In conclusion, homocysteine concentration is associated with SBP and DBP in both men and women in conventional regression analysis. MR analyses do not support a causal role of homocysteine in SBP in young southern Brazil adults, but results for DBP were less conclusive. Findings from older adults (ICBP data) corroborated the results for SBP, but it was not possible to rule out a causal, positive effect of genetically instrumented homocysteine on DBP; however, this association was largely driven by a single SNP and was not consistent across other SNPs associated with homocysteine. Overall, our findings indicate that homocysteine is more likely a marker than a cause of increased blood pressure, especially for SBP.

## Footnotes

5 Abbreviations used: CBS, cystathionine β-synthase; CVD, cardiovascular disease; DBP, diastolic blood pressure; DWH, Durbin-Wu-Hausman; GIANT, Genetic Investigation of Anthropometric Traits; GLGC, Global Lipids Genetics Consortium; GWAS, genomewide association study; HbA1c, glycated hemoglobin; ICBP, International Consortium for Blood Pressure; IV, instrumental variable; IVW, inverse variance weighted; MAGIC, Meta-Analyses of Glucose and Insulin-Related Traits Consortium; MR, Mendelian randomization; MTHFR, methylenetetrahydrofolate reductase; OLS, ordinary least-squares; SBP, systolic blood pressure; SNP, single nucleotide polymorphism; WC, waist circumference; 2SLS, 2-stage least-squares.
